# A *Peniophora lycii* Isolate Simultaneously Parasitizes *Vitis vinefera* Host and Associated Fungi, and Possibly Contributes to Grapevine Trunk Disease Development

**DOI:** 10.3390/jof12050348

**Published:** 2026-05-07

**Authors:** Nikolett Molnár, Dóra Szabó, Adrienn Gomba-Tóth, Ádám Novák, Kálmán Zoltán Váczy, Zoltán Karácsony

**Affiliations:** Food and Wine Research Institute, Eszterházy Károly Catholic University, Leányka Utca 8/G, H-3300 Eger, Hungary; molnar.nikolett@uni-eszterhazy.hu (N.M.); szabo.dora@uni-eszterhazy.hu (D.S.); novak.adam@uni-eszterhazy.hu (Á.N.); vaczy.kalman@uni-eszterhazy.hu (K.Z.V.); karacsony.zoltan@uni-eszterhazy.hu (Z.K.)

**Keywords:** mycoparasitism, basidiomycete, trunk diseases, white rot

## Abstract

An isolate of *Peniophora lycii* was obtained from grapevine, and its interactions with several grapevine-associated fungi and the plant host were examined. The fungus was not able to infect intact leaves, but necrotized the margins of foliar disks and caused necrosis and white rot in woody tissues. In dual cultures, *P. lycii* and *Aureobasidium pullulans* showed mutual antagonism. Mycoparsitism of *P. lycii* was observed on epiphytic *Botrytis cinerea*, *Alternaria* sp., and endophytic *Botryosphaeria dothidea* interaction partners. In contrast, *P. lycii* showed trophic growth towards endophytic *Phaeomoniella chlamydospora* without any signs of harmful interactions. Dual inoculations of foliar disks with epiphytic fungi revealed no effects of fungal interactions on necrosis development by pathogens and verified mycoparasitic interactions in planta. Co-infection assays of cuttings with endophytic pathogen fungi showed cumulative effects of fungal interactions on wood symptom expression, with the exclusive contribution of *P. lycii* to white rot development. In addition to providing the first isolation of *P. lycii* from grapevine and the description of its mycoparasitic behavior, the present study suggests that the fungus may act as an opportunistic grapevine pathogen, probably as a secondary colonizer in trunk diseases. The observed dual host preference may allow trunk disease pathogens to initially feed on fungi, followed by damage to the grapevine. This may be in connection with the exceptionally long latency of these syndromes.

## 1. Introduction

*Peniophora lycii* [1], a member of the Peniophoraceae family within the order Russales, is a white-rot, wood-decaying basidiomycete commonly found on dead branches and the stems of woody plants such as *Fraxinus excelsior* [2], *Quercus robur* [3], *Corylus avellana*, *Abies*, *Juniperus*, *Larix*, *Picea*, *Pinus*, and *Acer* spp. [4]. As a saprotrophic fungus, it plays an essential role in forest ecosystems by decomposing lignocellulosic materials, particularly cellulose, thereby contributing significantly to nutrient cycling [5]. White rot fungi have the capability to directly or indirectly oxidize lignin via an enzymatic system. This system contains enzymes such as lignin peroxidase, manganese peroxidase, and laccase [6]. According to a previous study, *P. lycii* produces laccases, lytic polysaccharide monooxygenases, carbohydrate-active enzymes, and oxidoreductases. Their activity results in the production of H_2_O_2_ and phenol radicals that depolymerize lignin, cellulose, and hemicellulose particles [5].

The complex lignolytic machinery evolved in basidiomycetous white rot fungi clearly associates them with plants. However, this association can be expressed in different ecological roles. Although these fungi are mostly recognized as decomposers of dead plant biomass in forest ecosystems [7], a phytopathogenic lifestyle also occurs in this group. This dual role can also be observed in closely related species such as the saprophytic *Armillaria cepistipes* and the pathogenic *Armillaria ostoyae*, both colonizing spruce [8]. In addition, several species of wood-decaying fungi are capable of both decomposing dead wood and infecting a living host. This adaptation is possibly achieved by gene expression reprogramming, as reported previously in the case of *Heterobasidion irregulare* [9]. This behavioral plasticity suggests that saprophytic basidiomycetes can easily adapt to a pathogenic lifestyle, posing a threat to agriculture. Fungal white rot diseases were previously reported from several woody crops, including citrus [10], kiwi [11], and olive trees [12].

Grapevine hosts a wide range of microorganisms, including several fungal species. Both grapevine leaf and wood mycobiomes are dominated by ascomycetous fungi, whereas basidiomycetes are less abundant [13,14]. Among these basidiomycetous species, several white-rot fungi can be found, such as *Arambarria cognata* [15], *Inocutis jamaicensis* [16], and *Phellinus igniarius* [17]. An increased abundance of basidiomycetes in grapevine wood is associated with the development of grapevine trunk diseases (GTDs) [18]. These syndromes are devastating fungal infections of grapevine. The causal agents infect the grapevine wood, causing necrosis in the xylem, followed by the development of symptoms on the annual parts, and eventually host death [19]. Although most of the related pathogens are ascomycetous fungi, some basidiomycetes have also been found to be associated with GTD development, such as *Fomitiporia mediterranea* in the case of Esca syndrome [20]. Other basidiomycetous species, such as *Stereum hirsutum*, *Trametes hirsuta*, and *Schizophyllum commune*, are referred to as secondary colonizers in wood previously damaged by ascomycetous GTD pathogens [21].

The present study characterizes the first isolate of *P. lycii* obtained from grapevine. According to phytopathogenicity tests, the examined isolate could be a wound parasite on the annual parts and may contribute to GTD development by causing white rot in the wood. In addition, the fungus showed mycoparasitism toward several grapevine-associated fungi, both in vitro and in planta, with no preference to either the grapevine or fungal hosts.

## 2. Materials and Methods

### 2.1. Strains and Growth Conditions

The fungal strains used in the present study are listed in Table 1. Fungi were routinely cultivated on potato dextrose agar (PDA; Merck KGaA, Darmstadt, Germany) medium and cryopreserved at −80 °C, in 50% *v*/*v* glycerol.

### 2.2. Isolation and Identification of Fungal Strains

Grapevine leaf samples were collected from Cabernet Sauvignon cultivar, in Eger, Hungary (47.868477 N; 20.387326 E), from healthy grapevines. Sampling was performed on 5 September 2024. To preserve epiphyllous fungal communities, the leaves were aseptically collected and stored in sterile bags until further processing. Foliar disks with a 2.5 cm diameter were cut and vigorously rinsed with 5 mL of distilled sterile water. A volume of 25 µL of the collected wash solution was spread onto DRBC agar (dichloran, rosebengal, chloramphenicol) and incubated at 25 °C in the dark under continuous monitoring. Fungi overgrowing neighboring colonies as potential mycoparasites were selected and subcultured for further investigations.

Total DNA was extracted from the fungal biomass for molecular identification using a DNeasy Plant Mini Kit (Qiagen, Venlo, The Netherlands) according to the manufacturer’s instructions. Internal transcribed spacer (ITS) regions were amplified by using ITS1-f (5′-CTTGGTCATTTAGAGGAAGTAA-3′) and ITS4 (5′-TCCTCCGCTTATTGATATGC-3′) primers. Partial ribosomal large-subunit rDNA (LSU) was amplified by LR5 (5′-TCCTGAGGGAAACTTCG-3′) and LR0R (5′-ACCCGCTGAACTTAAGC-3′) primers. PCR reactions were carried out under the following conditions: denaturation at 95 °C for 45 s; annealing at 52 °C (ITS) or 55 °C (LSU) for 45 s; polymerization at 72 °C for 60 s (ITS) or 75 s (LSU). Both reactions were performed with 32 cycles. To check the PCR reaction efficiency, agarose gel electrophoresis was performed. The amplicons were sequenced (Microsynth Seqlab GmbH, Göttingen, Germany), and the sequences were compared in online databases using BLAST (https://www.ncbi.nlm.nih.gov). The obtained sequences were used for phylogenetic analysis using the MEGA12 (v12.0.9) program [25]. The alignment of the sequences was performed via Clustal W [26], and the phylogeny was inferred using the maximum likelihood method and Tamura–Nei model [27] of nucleotide substitutions. The tree with the highest log likelihood (−7025.33) was established by the MEGA12 program. The sequences used in the phylogenetic analysis are listed in Supplementary Table S1.

### 2.3. Morphological Observations

One-centimeter-wide mycelial disks were cut from the edge of one-week-old fungal colonies growing on PDA and inoculated in the center of PDA, malt extract agar (MEA; Merck KGaA, Darmstadt, Germany), oatmeal agar (OA; Merck KGaA, Darmstadt, Germany), and 2% *w*/*v* water agar (WA). Cultures were incubated at 25 °C in the dark for 14 days and photographed. All inoculations were performed in triplicate.

### 2.4. Detection of Secreted Degradative Enzymes

The secretion of digestive enzymes by the examined *P. lycii* strain was investigated by the use of indicative media. Czapek dox media [28] containing 2% *w*/*v* sucrose, 0.2% *w*/*v* NaNO_3_, 0.1% *w*/*v* K_2_HPO_4_, 0.05% *w*/*v* MgSO_4_, 0.05% *w*/*v* KCl, 0.001% *w*/*v* FeSO_4_, 2% *w*/*v* agar were prepared and supplemented with different substrates for the different exoenzymes. Carboxymethylcellulose (1% *w*/*v*) was used for cellulase, water-soluble starch (1% *w*/*v*) for amylase, and ABTS (2 mM) for laccase activity detection [29].

The examined *P. lycii* isolate was inoculated onto the indicative media as mycelial plugs with a 3 mm diameter, pre-grown on PDA medium. The effects of the observed digestive enzymes were detected after 7 days of incubation at 25 °C. Staining with Gram’s iodine (0.33% *w*/*v* iodine and 0.67% *w*/*v* potassium iodide) was carried out for amylase and cellulase visualization. The detection of laccase activity was performed by the observation of green-–blue colorization of colonies [29]. All inoculations were performed in triplicate.

### 2.5. In Vitro Dual Culture Assay

To study the possible interactions of *P. lycii* isolate with *B. cinerea*, *Alternaria* sp., *B. dothidea*, *P. chlamydospora*, and *A. pullulans*, dual culture assays were performed using the strains listed in Table 1. Mycelial plugs (3 mm diameter) were cut from the margins of 1-week-old colonies grown on PDA and placed on PDA, Czapek dox medium, as well as WA covered with cellophane. Interaction partners were inoculated at a distance of 4–5 cm from each other. The petri dishes were incubated at 25 °C in the dark for a maximum of two weeks and monitored daily. All inoculations were performed in triplicate.

### 2.6. Phytopathogenicity Tests

#### 2.6.1. Artificial Infection of Detached Leaves

Fully expanded detached leaves of one-year-old Cabernet Sauvignon cuttings were immersed in 70% (*v*/*v*) ethanol for 30 s, then extensively washed with sterile distilled water. After five washing cycles, the leaves were placed on the surface of WA plates, with their petiole inserted into the agar. The examined *P. lycii* isolate was pre-grown on WA for one week, and mycelial plugs of about 1 × 1 cm were cut and placed on the adaxial surface of detached leaves. Sterile WA plugs were used as a negative control. The inoculations were carried out in triplicate, and the leaves were incubated at 21 ± 2 °C with a 12 h photoperiod under a fluorescent lamp (6500 K color temperature) for seven days.

#### 2.6.2. Artificial Infection of Foliar Disks

To ensure the reproducibility of artificial infections and in planta microbial interaction assays with epiphytes, as well as the accuracy of the measurement of foliar necrosis, foliar disk assays were carried out. Grapevine foliar disks with a 1 cm diameter were cut from fully expanded and healthy leaves of one-year-old Cabernet Sauvignon cuttings. Leaves were collected from the third level from the basal leaf by removing one leaf from five cuttings. The disks were pooled and were disinfected by immersing in 70% *v*/*v* ethanol for 30 s, followed by extensive washing with sterile distilled water five times. Thereafter, the disks were briefly dried, and five of them were placed at the central line of 9 cm diameter plates on top of WA. Fungal strains were inoculated as one-week-old PDA mycelial plugs at a distance of 1 cm from the nearest foliar disk. For co-inoculation experiments, different fungal strains were inoculated on the two sides of the disks. Uninoculated plates were also prepared as a control. Foliar disks were incubated at 21 ± 2 °C with a 12 h photoperiod under a fluorescent lamp (6500 K color temperature) for seven days.

#### 2.6.3. Artificial Infection of Cuttings

One-year-old Cabernet Sauvignon cuttings were used to assess fungal pathogenicity on wounded grapevine wood. Cuttings were drilled and inoculated with the fungal strains alone or in combination with confrontation partners as 3 mm wide mycelial plugs, cut from colonies pre-grown on PDA for a week. Mock inoculations were performed by placing sterile PDA plugs into the wounds. All types of inoculations were carried out in five replicates on five different cuttings. The inoculated wounds were sealed with parafilm, and the plants were incubated in the greenhouse without artificial lighting and with full exposure to sunlight. The greenhouse lacked precise temperature control, but the top windows automatically opened on hot days to prevent heat stress on plants. Incubation was performed for 90 days. Cuttings were inoculated in the middle of May and processed in the middle of August. This late-summer period is also the peak of grapevine trunk disease foliar symptom expression in the field in Hungary. The cuttings were uprooted, and the bark was removed to observe necrosis developing on the xylem surface. Longitudinal sections were prepared to observe inner necrosis and white rot inside the canes. To re-isolate the inoculated fungi, disks were cut 5–10 mm above the inoculation points. The pieces were surface-sterilized in sodium hypochlorite (4% *w*/*v* chlorine) for 2 min, followed by incubation in 70% *v*/*v* ethanol for 2 min. After drying, the sterilized disks were cut into three pieces and placed on PDA medium. Cultures were incubated at 25 °C for one week. Emerging mycelia were transferred to fresh PDA plates. After an initial morphological screening, selected pure cultures were used for DNA extraction and PCR analyses to verify the identity of the re-isolated fungi according to the ITS sequence, as described in Section 2.2.

### 2.7. Microscopic Examinations

To study the hyphal interactions of the examined fungal strains, small pieces were cut from the cellophane in the confrontation zones on WA dual cultures prepared as described above. Samples were mounted in distilled water, covered with a coverslip, and examined.

To examine *P. lycii* mycelia growing on plant tissues, foliar disk inoculations were prepared as described above. Disks were immersed in 70% *v*/*v* ethanol for 5 min, followed by staining with calcofluor white solution (50 µg/mL in distilled water) for 5 min. Stained disks were briefly washed with distilled water and placed in a drop of 50% *v*/*v* glycerol on a glass slide and observed.

For the investigation of hyphal interactions between fungal strains on artificially infected foliar disks, inoculations were prepared as described above. The surface of colonized plant material was sampled by the use of sellotape. Samples were briefly stained with lactophenol cotton blue, covered with a coverslip, and examined.

All microscopic observations were performed using an Olympus BX53F2 (Olympus Corporation, Tokyo, Japan) microscope equipped with a phase contrast optical accessory and a DP47 camera. Fluorescent dye was excited by an LED light source (λ = 360–665 nm). To observe the blue fluorescence of calcofluor white, a filter set with λex = 360–370 nm, and λem = 420–460 nm was used.

### 2.8. Software

Statistical comparisons were performed using GraphPad Prism 5 software (GraphPad Software, San Diego, CA, USA, https://www.graphpad.com) using one-way ANOVA with Tukey’s post hoc test. For image analysis, the Fiji software (v20250529-2217) was used [30]. Photomicrographs were taken using CellSens Entry (v4.1) software (Olympus Corporation, Tokyo, Japan).

## 3. Results

### 3.1. Identification and Morphological Characterization of Fungal Isolates

Isolate BCA32024 was identified as *Alternaria* sp. according to ITS sequencing (GenBank accession: PX945795). For phylogenetic analyses of the BCA72024 isolate, two genomic loci were used (ITS, GenBank accession: PX957018; LSU, GenBank accession: PX963637). The ITS sequence clearly linked the isolate to the *Peniophora* genus, whereas the LSU showed the highest similarity to a *P. lycii* strain (strain CBS352.54, GenBank accession: MH868899.1, 99.89% identity). Multi-locus phylogenetic analysis of the ITS + LSU sequences further reinforced the identity of isolate BCA72024 as *P. lycii* (Supplementary Figure S1).

**Figure 1:**
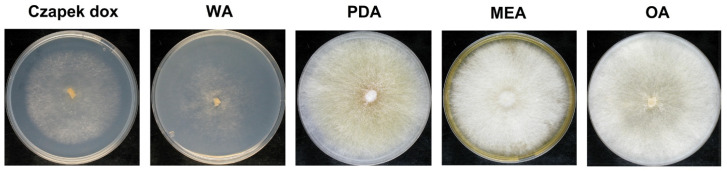
*P. lycii* BCA72024 colonies grown on Czapek dox, 2% *w*/*v* water agar (WA), potato dextrose agar (PDA), malt extract agar (MEA), and oatmeal agar (OA) at 25 °C, for 14 days.

Macromorphological observations of *P. lycii* BCA72024 on different media showed that the isolate formed colonies with even margins and white mycelia on all tested media, except on the PDA plate, on which *P. lycii* exhibited yellowish coloration (Figure 1). Microscopic examination of WA cultures showed thin-walled hyaline hyphae with clamps, which is in agreement with the micromorphological characteristics of the species [1].

### 3.2. In Vitro Detection of Secreted Degradative Enzymes of P. lycii

**Figure 2:**
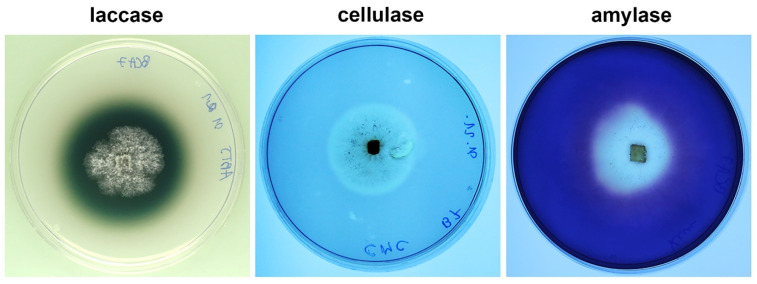
Detection of exoenzyme activity of *P. lycii* BCA72024 for laccases, cellulases, and amylases on Czapek dox media after 7 days of incubation at 25 °C. Media were supplemented with 2 mM ABTS for laccases, with 1% *w*/*v* carboxymethyl cellulose for cellulases and 1% *w*/*v* starch for amylases. Clearance zones were detected by staining with Gram’s iodine in the case of cellulases and amylases.

The examined *P. lycii* isolate produced detectable amounts of laccases, cellulases, and amylases in vitro, according to experiments carried out on indicative media (Figure 2). There were considerable differences between the observed enzyme activities. Although laccase activity was expressed at a high level (22.18 ± 0.89 cm^2^ activity zone area), the cellulase-indicating halo around the colonies was narrow (8.70 ± 1.28 cm^2^ zone of clearance), and amylase activity was restricted to the center of the colonies (6.12 ± 1.46 cm^2^ zone of clearance).

### 3.3. Investigation of the Effects of P. lycii on Grapevine

**Figure 3:**
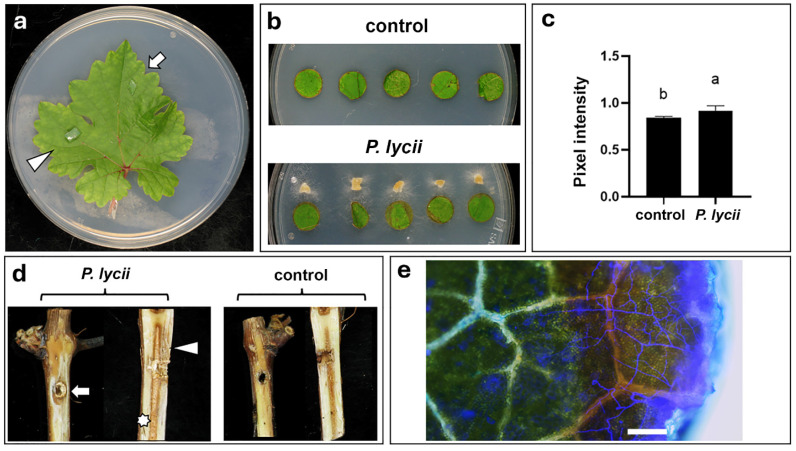
Effect of *P. lycii* BCA72024 inoculations on grapevine. (**a**) Detached grapevine leaf inoculated with *P. lycii* PDA mycelial plug (white arrow) or sterile PDA medium (white arrowhead) after 7 days of incubation. (**b**) Grapevine foliar disks on WA infected or left uninoculated (control) for 7 days. (**c**) Rate of red/green pixel intensity of inoculated and uninoculated (control) foliar disks. Letters mark significantly differing (*p* < 0.05) datasets. (**d**) Debarked surface and longitudinal sections of wounded grapevine cuttings artificially infected, or left uninoculated (control) for 90 days. White arrow: surface necrosis of xylem, white arrowhead: inner necrosis of xylem, white asterisk: white necrosis of pith. (**e**) Merged bright field and blue fluorescence photomicrographs of foliar disk incubated on WA for 7 days after infection and subsequent calcofluor white staining. Scale bar: 100 µm.

On detached grapevine leaves, there was no observable pathogenic effect of the examined *P. lycii* isolate after 7 days of incubation (Figure 3a). In contrast, minor necrotic symptoms appeared at the edges of the inoculated foliar disks (Figure 3b). The ability of the fungus to necrotize the foliar disks was reinforced by the statistical analysis of red/green pixel intensity measurements (Figure 3c, Supplementary Table S2). Fluorescent staining and microscopic observations of inoculated foliar disks showed that the fungal growth was restricted to the necrotized edge of the infected plant material (Figure 3e).

On artificially infected grapevine cuttings, the examined *P. lycii* isolate caused the frequent development of dark discoloration on the xylem surface and inside the xylem (Figure 3d). However, only internal necrosis showed significantly higher severity in infected cuttings compared with uninoculated control plants (Supplementary Table S3). White rot of cane pith was more characteristic of *P. lycii* infections (Figure 3d); it occurred in three out of the five infected cuttings, while this symptom was totally absent in uninoculated plants (Supplementary Table S3). The *P. lycii* isolate could be re-isolated from all five infected cuttings.

### 3.4. Interactions of P. lycii and Epiphytic Fungi of Grapevine

#### 3.4.1. Interaction of *P. lycii* and *B. cinerea*

**Figure 4:**
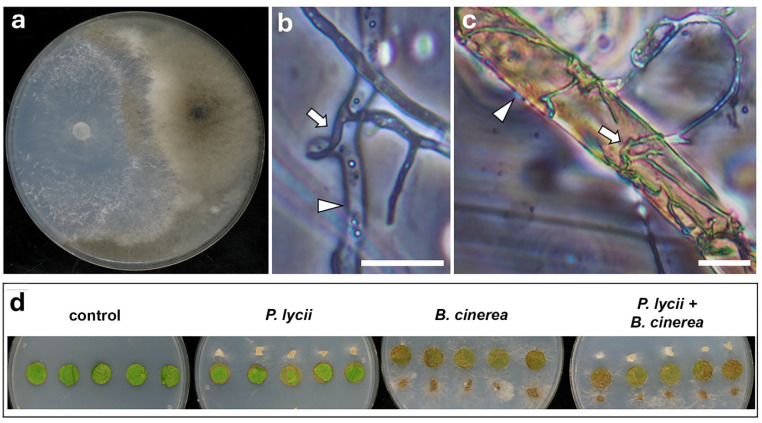
Interactions of *P. lycii* BCA72024 with *B. cinerea* Or13. (**a**) Confrontation test of *P. lycii* (**left**) and *B. cinerea* (**right**) colonies on PDA medium at 25 °C, after 7 days. (**b**) Phase contrast photomicrograph of interacting hyphae of *P. lycii* (white arrow) and *B. cinerea* (white arrowhead) grown on WA for 7 days. Scale bar: 10 µm. (**c**) Phase contrast photomicrograph of interacting hyphae of *P. lycii* (white arrow) and *B. cinerea* (white arrowhead) sampled from co-inoculated grapevine foliar disks after 7 days. Scale bar: 50 µm. (**d**) Grapevine foliar disks inoculated with *P. lycii* and/or *B. cinerea*, or left uninoculated (control) after 7 days incubation on WA. Control is the same as that in Figure 3b.

In the in vitro dual culture assay, the *P. lycii* isolate overgrew the surface of the interacting *B. cinerea* colony (Figure 4a). The mycoparasitic interaction was verified by the microscopic examination of WA dual cultures (Figure 4b) and co-inoculated foliar disks (Figure 4c), showing the intracellular growth of *P. lycii* in *B. cinerea* mycelia. In both cases, the colonized *B. cinerea* hyphae seemed to be empty, indicating cell death. Foliar disk inoculations (Figure 4d) showed that *B. cinerea* had a detrimental effect on the condition of plant tissues, whereas *P. lycii* caused limited necrosis, as mentioned above. Co-inoculation of foliar disks resulted in the same extent of necrosis as sole *B. cinerea* inoculation, according to image analysis (Figure 4d, Supplementary Table S2).

#### 3.4.2. Interaction of *P. lycii* and *Alternaria* sp.

**Figure 5:**
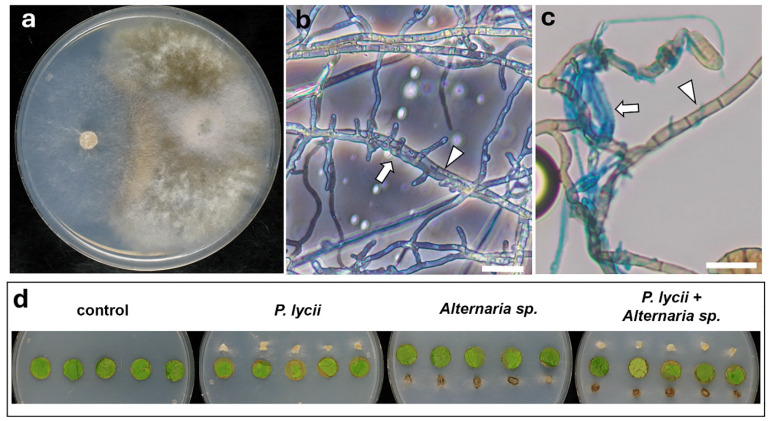
Interactions of *P. lycii* BCA72024 with *Alternaria* sp. BCA32024. (**a**) Confrontation test of *P. lycii* (**left**) and *Alternaria* sp. (**right**) colonies on PDA medium at 25 °C, after 7 days. (**b**) Phase contrast photomicrograph of interacting hyphae of *P. lycii* (white arrow) and *Alternaria* sp. (white arrowhead) grown on WA for 7 days. Scale bar: 20 µm. (**c**) Phase contrast photomicrograph of interacting hyphae of *P. lycii* (white arrow) and *B. cinerea* (white arrowhead) sampled from co-inoculated grapevine foliar disks after 7 days. Scale bar: 20 µm. (**d**) Grapevine foliar disks inoculated with *P. lycii* and/or *Alternaria* sp., or left uninoculated (control) after 7 days of incubation on WA. Control is the same as in Figure 3b.

Similar to *B. cinerea*, colonies of *Alternaria* sp. were overgrown by the tested *P. lycii* isolate in the PDA dual cultures (Figure 5a). Extensive external colonization of the host hyphae by *P. lycii* was observed on WA dual cultures (Figure 5b), as well as on co-inoculated grapevine foliar disks (Figure 5c). This phenomenon was accompanied by signs of death on affected *Alternaria* sp. cells. In foliar disk inoculation assays, the presence of *Alternaria* sp. did not affect the necrosis caused by *P. lycii* according to image analysis (Figure 5d, Supplementary Table S2).

#### 3.4.3. Interaction of *P. lycii* and *A. pullulans*

**Figure 6:**
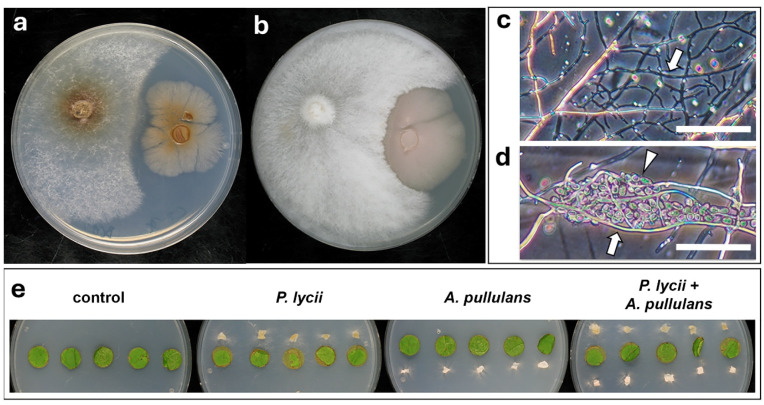
Interactions of *P. lycii* BCA72024 with *A. pullulans* Y37. (**a**) Confrontation test of *P. lycii* (**left**) and *A. pullulans* (**right**) colonies on PDA medium at 25 °C, after 7 days. (**b**) Confrontation test of *P. lycii* (**left**) and *A. pullulans* (**right**) colonies on Czapek dox agar medium at 25 °C, after 7 days. (**c**) Phase contrast photomicrograph of *P. lycii* hyphae (white arrow) near an *A. pullulans* colony on WA at 25 °C, after 7 days. (**d**) Phase contrast photomicrograph of *P. lycii* hyphae (white arrow) and *A. pullulans* yeast cells (white arrowhead) on WA at 25 °C, after 7 days. (**e**) Grapevine foliar disks inoculated with *P. lycii* and/or *A. pullulans*, or left uninoculated (control), after 7 days incubation on WA. Control is the same as that in Figure 3b.

The interaction of *A. pullulans* with *P. lycii* differed notably from the above-observed mycoparasitic interactions against *B. cinerea* (Figure 4) and *Alternaria* sp. (Figure 5). On Czapek–Dox agar, *A. pullulans* exhibited strong antibiotic activity against *P. lycii* (Figure 6a). In contrast, the *P. lycii* isolate showed dominance on PDA medium, but without overgrowth on the colony of the confrontation partner (Figure 6b). Microscopically, both fungi showed signs of stress in WA dual cultures. The *P. lycii* mycelia were mostly empty near the interaction partner colony (Figure 6c) in accordance with the previously observed antibiosis of *A. pullulans* against *P. lycii* (Figure 6a). However, a limited number of *P. lycii* hyphae were able to reach the *A. pullulans* cells, which latter showed yeast morphology under the applied experimental condition. In these cases, *P. lycii* hyphae colonized the *A. pullulans* cells. These latter often showed a collapsed appearance, suggesting cell death (Figure 6d). Microscopic study of the interactions of the fungal confrontation partners could not be done on foliar disks, as *A. pullulans* was again grown in its yeast form, hampering the fixation of cell–cell interaction structures during sample preparation. Foliar disk inoculations did not show any damage caused by *A. pullulans* (Figure 6e). The presence of this potent antagonist surprisingly did not significantly affect the severity of necrosis caused by *P. lycii* according to image analysis (Figure 6e, Supplementary Table S2).

### 3.5. Interactions of P. lycii and Endophytic GTD Pathogens

#### 3.5.1. Interaction of *P. lycii* and *P. chlamydospora*

**Figure 7:**
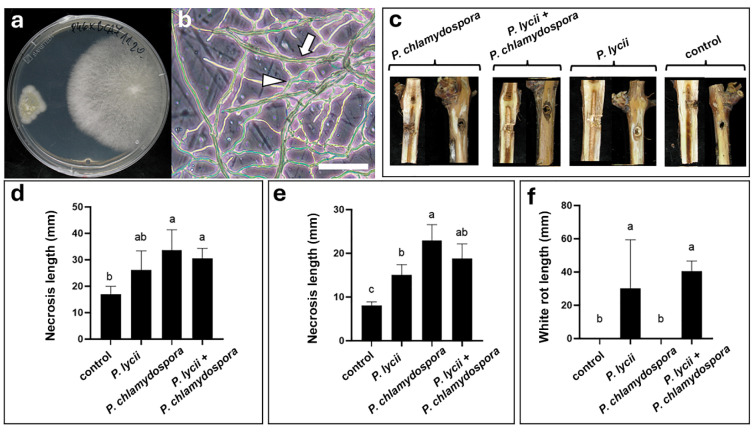
Interactions of *P. lycii* BCA72024 with *P. chlamydospora* P46. (**a**) Confrontation test of *P. lycii* (**right**) and *P. chlamydospora* (**left**) colonies on PDA medium at 25 °C, after 7 days. (**b**) Phase contrast photomicrograph of interacting hyphae of *P. lycii* (white arrow) and *P. chlamydospora* (white arrowhead) grown on WA at 25 °C, after 7 days. Scale bar: 50 µm. (**c**) Photographs of the surface and longitudinal section of debarked cuttings inoculated with *P. lycii* and/or *P. chlamydospora*, or left uninoculated (control), after 90 days of incubation. Control is the same as that in Figure 3d. (**d**) Mean length and standard deviation of external necrosis on cuttings inoculated with *P. lycii* and/or *P. chlamydospora*, or left uninoculated (control), after 90 days of incubation. (**e**) Mean length and standard deviation of internal necrosis in cuttings inoculated with *P. lycii* and/or *P. chlamydospora*, or left uninoculated (control), after 90 days of incubation. (**f**) Mean length and standard deviation of white rot in cuttings inoculated with *P. lycii* and/or *P. chlamydospora*, or left uninoculated (control), after 90 days of incubation. Letters on graphs indicate significantly differing (*p* < 0.05) datasets.

In the dual culture assays, *P. lycii* exhibited tropism toward *P. chlamydospora* on PDA medium (Figure 7a). Statistical analysis reinforced that *P. lycii* showed a significantly higher growth rate toward *P. chlamydospora* colonies (Supplementary Table S4). Microscopic examination of dual cultures on WA did not reveal any harmful effects of the confrontation partners on each other (Figure 7b). Mycelia of both species grew together without any observable hyphal interactions or signs of stress. Phytopathogenicity tests on cuttings showed that external and internal necrosis of the xylem could be observed in all inoculated plants, whereas white rot developed only in cuttings infected with *P. lycii* (Figure 7c, Supplementary Table S3). The severity of external (Figure 7d, Supplementary Table S3), and internal necrosis (Figure 7e, Supplementary Table S3) showed an insignificant decrease in co-inoculated plants compared with cuttings inoculated solely with *P. chlamydospora*. There was no difference in white rot severity between plants inoculated solely with *P. lycii*, or those co-infected with *P. lycii* and *P. chlamydospora* (Figure 7f, Supplementary Table S3). The *P. lycii* strain could be re-isolated from all five co-infected cuttings.

#### 3.5.2. Interaction of *P. lycii* and *B. dothidea*

**Figure 8:**
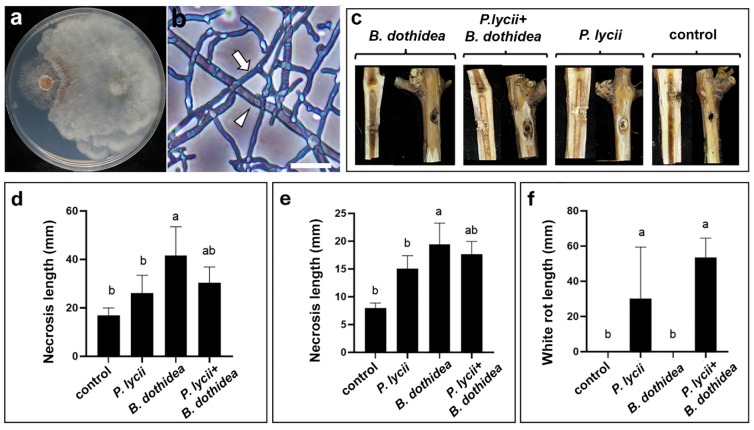
Interactions of *P. lycii* BCA72024 with *B. dothidea* 99C1. (**a**) Confrontation test of *P. lycii* (**left**) and *B. dothidea* (**right**) colonies on PDA medium at 25 °C, after 7 days. (**b**) Phase contrast photomicrograph of interacting hyphae of *P. lycii* (white arrow) and *B. dothidea* (white arrowhead) grown on WA at 25 °C, after 7 days. Scale bar: 50 µm. (**c**) Photographs of the surface and longitudinal section of debarked cuttings inoculated with *P. lycii* and/or *B. dothidea*, or left uninoculated (control), after 90 days of incubation. Control is the same as that in Figure 3d. (**d**) Mean length and standard deviation of external necrosis on cuttings inoculated with *P. lycii* and/or *B. dothidea*, or left uninoculated (control) after 90 days of incubation. (**e**) Mean length and standard deviation of internal necrosis in cuttings inoculated with *P. lycii* and/or *B. dothidea*, or left uninoculated (control), after 90 days of incubation. (**f**) Mean length and standard deviation of white rot in cuttings inoculated with *P. lycii* and/or *B. dothidea*, or left uninoculated (control), after 90 days of incubation. Letters on graphs indicate significantly differing (*p* < 0.05) datasets.

In the case of *B. dothidea*, *P. lycii* clearly overgrew the competing colony in the PDA dual culture assays (Figure 8a). Microscopic analysis revealed *P. lycii* hyphae tightly wrapping around the hyphae of *B. dothidea*, which latter showed an empty and pale appearance, suggesting cell death (Figure 8b). Observation of artificially infected cuttings revealed the presence of external and internal necrosis, as well as white rot in the inoculated plants (Figure 8c, Supplementary Table S3). Analysis of the pathogenicity test measurements showed that—similarly to *P. chlamydospore*—the severity of external (Figure 8d, Supplementary Table S3) and internal necrosis (Figure 8e, Supplementary Table S3) caused by *B. dothidea* insignificantly decreased by the co-inoculated *P. lycii*. White rot was only present in plants inoculated with *P. lycii*, and symptom severity was not affected significantly by the co-inoculation of *B. dothidea* (Figure 8f, Supplementary Table S3). The *P. lycii* strain could be re-isolated from all five co-inoculated cuttings.

## 4. Discussion

Although *P. lycii* is a wood-decaying basidiomycete and is commonly found on dead branches and stems of woody plants, to our knowledge, this is the first report of *P. lycii* isolated from grapevine. The *Peniophora* genus has previously been associated with the grapevine in epiphytic and endophytic microbiome studies [31]. *Peniophora* species, such as *Peniophora incarnata*, are considered saprophytes on grapevines in DNA metabarcoding analysis [32]. Other species, such as *Peniophora albobadia* [33] and *Peniophora crassitunicata* [34], have been tested in grape pomace biotransformation experiments focusing on lignocellulose degradation. *Peniophora cinerea* has been mentioned as a taxon found in grapevine samples in the Northern Midwest in 2019 [35]. According to the above studies, *Peniophora* species are present in grapevines worldwide, and most likely have a saprotrophic lifestyle.

Infection assays on detached leaves revealed the inability of the examined *P. lycii* isolate to infect healthy green tissues (Figure 3a), whereas the fungus significantly necrotized the wounded margin of the foliar disks (Figure 3b,c, Supplementary Table S2). This latter result suggests that, although the tested *P. lycii* isolate was incapable of infecting healthy plant tissues, it exhibited phytotoxic activity. In addition, microscopic examinations revealed that the growth of the fungus was restricted to the previously necrotized tissues on the foliar disks (Figure 3c). The above results suggest that *P. lycii* could not infect the annual parts of the plant directly, but could grow in the wounds, leading to further necrotization of surrounding living host tissues and subsequent colonization. The molecular background of the ability of the examined *P. lycii* isolate to necrotize living host tissues is unclear, as there is no report on putative phytotoxins produced by either *P. lycii* or any members of the *Peniophora* genus.

The examined interactions of *P. lycii* with important members of the grapevine phyllosphere mycobiome suggest its complicated relationships with epiphytic fungi. *Botrytis cinerea* was selected for the experiments as the most relevant necrotrophic pathogen of grapevine [36], whereas *Alternaria* spp. are abundantly present on grapevine leaves without a clear association with diseases [37]. *Aureobasidium pullulans* is also a dominant member of the epiphytic mycobiome of grapevine, with strong antagonistic activity against several fungi [38]. in vitro confrontation assays clearly demonstrated the mycoparasitic behavior of the examined *P. lycii* isolate against both *B. cinerea* (Figure 4a–c) and *Alternaria* sp. (Figure 5a–c). The infected host hyphae were often empty, suggesting the necrotrophic nature of *P. lycii* mycoparsitism. To the best of our knowledge, this is the first observation of mycoparasitism in the genus *Peniophora*. Co-inoculation assays and subsequent microscopic examinations also detected these mycoparasitic interactions on foliar disks, suggesting that this phenomenon may also appear in field conditions. However, the presence of *Alternaria* sp. did not affect the necrosis on foliar disks caused by *P. lycii*, nor did *P. lycii* affect the damage caused by *B. cinerea* on leaf disks (Figure 4d and Figure 5d, Supplementary Table S2). These suggest that the mycoparasitism of the tested *P. lycii* isolate may show a low extent in planta, and it is independent of the phytotoxicity of the fungus. The interactions of *P. lycii* with the antagonist *A. pullulans* were controversial. On rich medium, *P. lycii* overgrew the *A. pullulans* colonies (Figure 6b), whereas *A. pullulans* showed a strong antibiotic effect on *P. lycii* on mineral medium (Figure 6a). This mutual antagonism was also observed in microscopic examinations: several *P. lycii* cells died near the *A. pullulans* colonies, whereas *A. pullulans* yeast cells were sometimes colonized and killed by *P. lycii* hyphae in WA dual cultures (Figure 6c). The controversial results of the dual culture assays on different media indicated that the outcome of the interaction between *P. lycii* and *A. pullulans* may depend on nutrient availability, with a higher antagonistic potential of *P. lycii* under nutrient-rich conditions. This suggests that damaged grapevine green tissues with high available nutrient levels are not just susceptible to *P. lycii* colonization, but also support its competing potential against antagonists, further reinforcing the wound-parasite lifestyle. In agreement with this hypothesis, the presence of *A. pullulans* did not affect the necrotizing ability of the tested *P. lycii* isolate on the damaged margins of foliar disks (Supplementary Table S2). It seems that the primary nutrient source for *P. lycii* under epiphytic growth conditions is the dead plant tissues, with a simultaneous utilization of fungal hosts. However, the damage caused to the grapevine or to the fungal hosts is limited. There is a lack of reports on the mycoparasitic behavior of fungi with a dominantly phytopathogenic lifestyle, with only some exceptions, such as the ascomycetous species *Microdochium majus* [22]. It may be a frequent but overlooked phenomenon, and the different host preferences may share some components of molecular machinery. This putative overlap between the two lifestyles requires further study.

Contrary to the grapevine leaves, the examined *P. lycii* isolate showed clear pathogenicity on the inoculated cuttings. The examined fungal strain caused the development of black necrosis under the bark and inside the xylem tissues, as well as extensive white rot in the cane pith (Figure 3d, Supplementary Table S3). These observations are in agreement with the ability of *P. lycii* isolate to produce extracellular enzymes degrading wood components (Figure 2). The fungus showed more expressed laccase activity compared with cellulase and amylase activities, suggesting its xylem-associated growth. This observed difference in enzyme activities is also in accordance with the higher severity of white rot symptoms compared with dark discolorations in *P. lycii*-infected cuttings, as laccases are important components in the wood-degrading enzymatic system of white rot fungi [6]. The observed wood damage in *P. lycii*-infected cuttings is the primary symptom of GTDs [19]. This suggests that *P. lycii* may be a member of the complex fungal community associated with these syndromes. In this context, *P. lycii* may act as a secondary colonizer. GTD-related basidiomycete fungi—such as *F. mediterranea* [20], or *S. hirsutum* [39]—colonize the host xylem in association with the damage caused previously by the ascomycetous primary pathogens (e.g., *B. dothidea* or *P. chlamydospora*) and develop white rot in the wood. In addition to their role in wood degradation, some recent studies suggest that basidiomycetes also contribute to the foliar symptom development of GTDs [40,41].

As the above results suggest that *P. lycii* is probably associated with GTDs, it was reasonable to study its interaction with GTD pathogens. For this purpose, *P. chlamydospora*, the main pathogen of Esca disease [42], and the Botryosphaeria dieback-related species *B. dothidea* [19] were used. The examined *P. lycii* strain showed necrotrophic mycoparasitism against *B. dothidea* (Figure 8a,b). Co-inoculation experiments on cuttings showed that this fungal interaction did not affect symptom severity, neither in the case of *B. dothidea*-related internal or external xylem necrosis, nor in the case of white rot in the cane pith caused by *P. lycii* (Figure 8c–f, Supplementary Table S3). These suggest that—as in the case of epiphytic interaction partners—*P. lycii* can simultaneously infect the plant and *B. dothidea*, with limited damage on the fungal host. Surprisingly, *P. lycii* mycelia showed trophic growth toward the colonies of *P. chlamydospora* in the in vitro confrontation assays (Figure 7a, Supplementary Table S4). There was no inhibitory effect observed when the colonies established physical contact, and the hyphae of both interaction partners did not show any interactions or any sign of damage (Figure 7b), suggesting that these two fungi can establish mixed colonies in vitro and possibly in planta. Infected grapevine cuttings showed no effect of co-inoculation on symptom severity, neither in the case of *P. chlamydospora*-related internal or external xylem necrosis, nor in the case of white rot in the can pith caused by *P. lycii* (Figure 7c–f, Supplementary Table S3). These results suggest that *P. lycii* and *P. chlamydospora* can simultaneously attack grapevines, resulting in the parallel development of necrosis and white rot, possibly leading to overall higher damage in co-infected hosts. This further reinforces the possibility that *P. lycii* may be part of GTD pathosystems, especially for esca disease. Growing evidence suggests that microbial interactions established by *P. chlamydospora* greatly affect esca disease development either in a negative [24] or a positive way [23]. A limited number of studies previously reported the presence of *Peniophora* species in GTD-affected grapevine. Studies based on DNA metabarcoding found that the members of *Peniophora* genus can be found in esca-symptomatic grapevines [40,43], and their abundance shows a correlation with botryosphaeria dieback pathogens of the *Neofusicoccum* and *Dothiorella* genera in planta [44]. *Peniophora incarnata* [45] and *P. cinerea* [35] were isolated from GTD symptomatic plants, but there has been no study examining the pathogenicity of *Peniophora* species in grapevine, nor their interactions with members of grapevine mycobiota.

Summarizing the above findings, *P. lycii* may act as an opportunistic wound parasite on annual parts of grapevine and as a white rot fungus in the woody tissues in association with GTD syndromes. It seems that, in parallel with the phytopathogenic behavior, *P. lycii* simultaneously parasitizes grapevine epiphytic and endophytic fungi. To the best of our knowledge, the present study is the first report of mycoparasitic behavior of a GTD-associated fungus. This suggests that some GTD pathogens can shift hosts during their development, partly explaining the long latency of these diseases. To verify the above-suggested ecological roles of *P. lycii*, additional strains need to be isolated from grapevine and subjected to characterization.

## Figures and Tables

**Table 1:** Fungal strains used in the present study.

Strain ID	Taxon	Loci Sequenced	Microbiome Role	Host	Year of Isolation	Reference
BCA72024	*Peniophora lycii*	ITS, LSU	this study	grapevine leaf	2024	This study
BCA32024	*Alternaria* sp.	ITS	epiphytic commensal	grapevine leaf	2024	This study
Or13	*Botrytis* *cinerea*	ITS	epiphytic pathogen	grapevine berry	2016	[22]
Y37	*Aureobasdidium* *pullulans*	ITS	antagonist	grapevine wood	2017	[23]
99C1	*Botryosphaeria* *dothidea*	ITS, EF	endophytic pathogen	grapevine wood	2018	[24]
P46	*Phaeomoniella* *chlamydospora*	ITS	endophytic pathogen	grapevine wood	2017	[23]

ITS: internal transcribed spacer, LSU: ribosomal large subunit rDNA, EF: transcription elongation factor 1-α gene.

## Data Availability

Nucleic acid sequences were uploaded to GenBank. Any other research data are presented in the study or as a Supplementary File.

## Supplementary Materials

The following supporting information can be downloaded at: https://www.mdpi.com/article/10.3390/jof12050348/s1, Figure S1: Multilocus phylogenetic tree of *P. lycii* BCA72024; Table S1: Strains and GenBank sequence accessions used in phylogenetic analysis; Table S2: Raw red/green pixel intensity values of foliar disk assays; Table S3: Raw symptom severity data of pathogenicity tests on cuttings; Table S4: Raw colony radius values of *P. lycii* BCA72024 in the presence or absence of *P. chlamydospora* P46.
