# Supplementary material for: A Peniophora lycii Isolate Simultaneously Parasitizes Vitis vinefera Host and Associated Fungi, and Possibly Contributes to Grapevine Trunk Disease Development
Source: J Fungi (Basel). 2026 May 7;12(5):348. doi: 10.3390/jof12050348 (PMC13208804; doi:10.3390/jof12050348)
Supplement: Supplementary file 1 [file jof-12-00348-s001.zip › Supplementary figure S1.pdf]

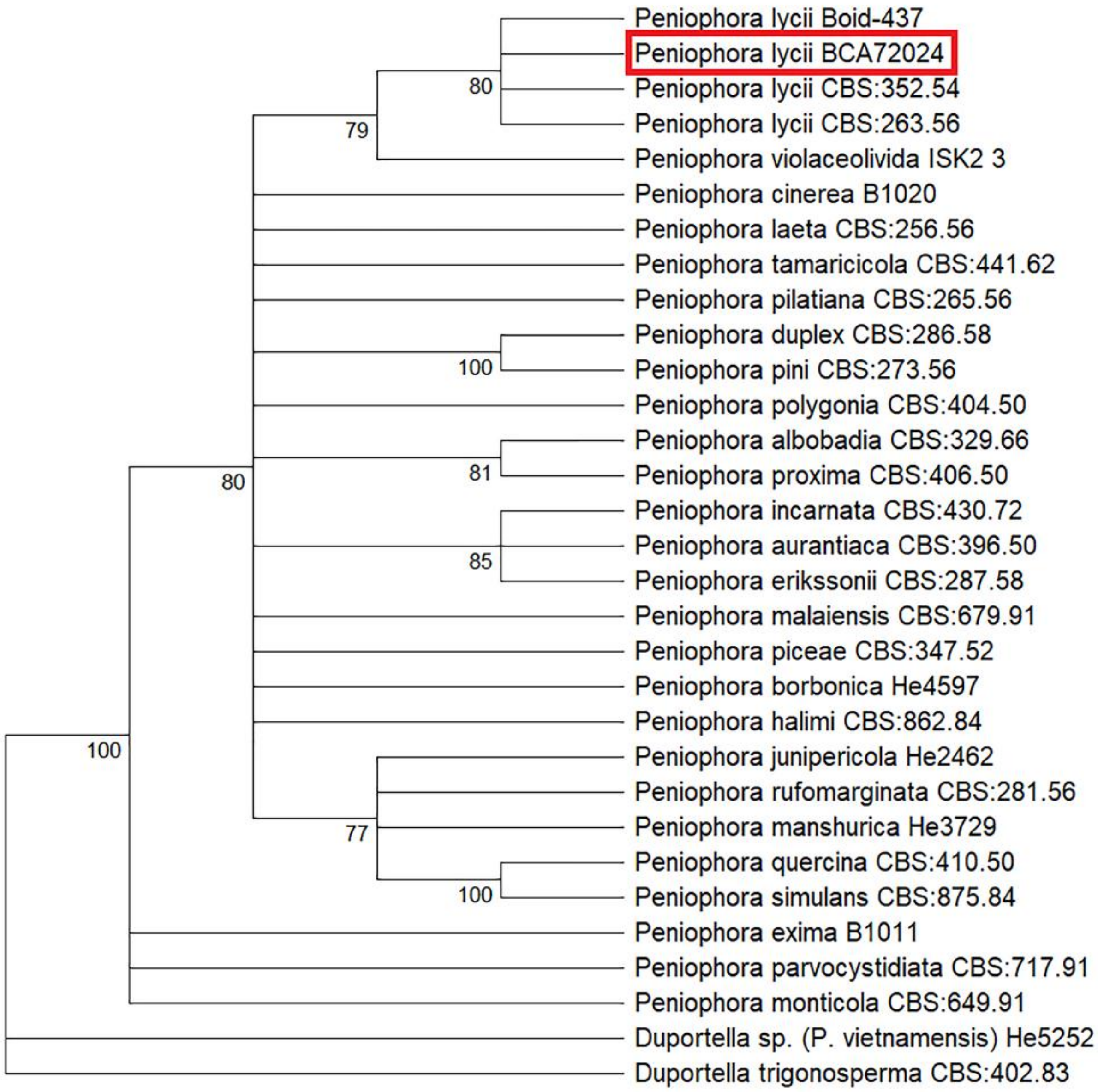

Figure 1. Maximum Likelihood analysis of two genes (ITS+LSU) combined dataset of *Peniophora* spp. using *Duportellas* spp. as an outgroup. Branches with a support value below 75% are collapsed. The fungal strain examined in this study is highlighted with a red box.
